# Genome-wide hydroxymethylation tested using the HELP-GT assay shows redistribution in cancer

**DOI:** 10.1093/nar/gkt601

**Published:** 2013-07-16

**Authors:** Sanchari Bhattacharyya, Yiting Yu, Masako Suzuki, Nathaniel Campbell, Jozef Mazdo, Aparna Vasanthakumar, Tushar D. Bhagat, Sangeeta Nischal, Maximilian Christopeit, Samir Parekh, Ulrich Steidl, Lucy Godley, Anirban Maitra, John M. Greally, Amit Verma

**Affiliations:** ^1^Cancer Center, Albert Einstein College of Medicine, 1300 Morris Park Avenue, Bronx, NY 10461, USA, ^2^Department of Genetics, Albert Einstein College of Medicine, 1300 Morris Park Avenue, Bronx, NY 10461, USA, ^3^Department of Pathology, Johns Hopkins School of Medicine, 1550 Orleans Street, Baltimore MD 21231, USA, ^4^Department of Medicine, University of Chicago, 5841 S. Maryland Avenue, Chicago, IL 60637, USA and ^5^Department of Medicine, Hematology and Medical Oncology, Mount Sinai School of Medicine, 1470 Madison Avenue, New York, NY 10029, USA

## Abstract

5-hydroxymethylcytosine (5-hmC) is a recently discovered epigenetic modification that is altered in cancers. Genome-wide assays for 5-hmC determination are needed as many of the techniques for 5-methylcytosine (5-mC) determination, including methyl-sensitive restriction digestion and bisulfite sequencing cannot distinguish between 5-mC and 5-hmC. Glycosylation of 5-hmC residues by beta-glucosyl transferase (β-GT) can make CCGG residues insensitive to digestion by MspI. Restriction digestion by HpaII, MspI or MspI after β-GT conversion, followed by adapter ligation, massive parallel sequencing and custom bioinformatic analysis allowed us determine distribution of 5-mC and 5-hmC at single base pair resolution at MspI restriction sites. The resulting HpaII tiny fragment Enrichment by Ligation-mediated PCR with β-GT (HELP-GT) assay identified 5-hmC loci that were validated at global level by liquid chromatography-mass spectrometry (LC-MS) and the locus-specific level by quantitative reverse transcriptase polymerase chain reaction of 5-hmC pull-down DNA. Hydroxymethylation at both promoter and intragenic locations correlated positively with gene expression. Analysis of pancreatic cancer samples revealed striking redistribution of 5-hmC sites in cancer cells and demonstrated enrichment of this modification at many oncogenic promoters such as *GATA6*. The HELP-GT assay allowed global determination of 5-hmC and 5-mC from low amounts of DNA and with the use of modest sequencing resources. Redistribution of 5-hmC seen in cancer highlights the importance of determination of this modification in conjugation with conventional methylome analysis.

## INTRODUCTION

The discovery of 5-hydroxymethylcytosine (5-hmC), an epigenetic modification of DNA, has led to studies that have shown that this chemical modification is prevalent in ES cells and tissues such as brain and kidney (1–3). The TET proteins have also been shown to be involved in the dioxygenation of 5-methylcytosine (5-mC) to 5-hmC residues (2,4). The discovery of mutations in TET proteins in various hematologic neoplasms also suggests that defects in 5-hmC pathway have functional consequences in carcinogenesis (5–8). In fact, deletion of *TET2* leads to hematopoietic alterations and neoplastic phenotypes in mice that are accompanied by concomitant decrease in total cellular 5-hmC (9). These studies highlight the need to study gene-specific localization of 5-hmC in the genome to understand how the distribution is altered in cancer.

Recently, TET-assisted bisulfite sequencing (10) and oxidative reduced representation bisulfite sequencing (11) have been described as assays that can analyze the hydroxymethylome at single base resolution, although these assays are dependent on great depth of sequencing restricting their utility in large-scale studies. An affinity-based method involving pull down of glycosylated 5-hmC residues has also been described, but requires large amounts of input DNA (12). To overcome these limitations we developed a high-throughput single base-pair resolution assay to identify 5-hmC and 5-mC in the genome by modifying our HELP-tagging assay. The HELP-tagging assay relies on the differential digestion of genomic DNA by HpaII and MspI enzymes (13). These are isoschizomers that act on the same CCGG sequence; while HpaII is methylation sensitive, MspI digests irrespective of CpG methylation status. By adding an extra glycosylation step before MspI digestion, we were able to interrogate both methylated and hydroxymethylated sites in the genome. This assay provides a genome-wide survey of both 5-hmC and 5-mC sites of the genome and can be used to analyze large sample cohorts with modest sequencing resources. Furthermore, we used this assay on pancreatic cancer samples and show for the first time that 5-hmC sites are widely redistributed in cancer.

## MATERIALS AND METHODS

### HELP-GT assay

Three hundred nanograms of genomic DNA were digested in three separate 100 µl reactions containing each of the following: A, beta-glucosyl transferase (β-GT)+MspI; B, MspI alone; and C, HpaII alone. To treat the DNA samples under similar conditions, reactions B and C were exposed at 37°C only with their respective buffers and UDPG, while only reaction A was exposed to 30 units of β-GT (New England Biolabs, MA, USA). Following a digestion of 8–9 h by β-GT, 30 units of MspI were added to A and B and 30 units of HpaII to C (for 16 h of digestion. Five microliters of the final digests were run on 1% agarose gel and the rest was purified by Puregene genomic DNA extraction kit (Qiagen). Eighty microliters of cell lysis solution and 60 µl of protein precipitation solution were added per 100 µl of digestion reaction. The tubes were inverted ten times and incubated on ice for 20 min and centrifuged at full speed for 10 min. The supernatant was incubated on ice for another 20 min and centrifuged. One microliter Ethachinamate was added to the final clear supernatant in a fresh tube and was precipitated with 160 μl isopropanol at −20°C for 16 h. Following centrifugation at full speed for 20 min, the pellet was washed twice with 70% ethanol, air-dried briefly and resuspended in 6 µl TE. Adapter EcoP15I side (AE adapter) ligation was performed in 13 µl reaction containing 2×Quick ligase buffer, 0.5 µl of 0.1 µM AE adapter, digested DNA and 1 µl of Quick Ligase (New England Biolabs, USA), for 15 min at reverse transcription. The following steps up to polymerase chain reaction (PCR) were done as previously (13) with some modifications in the AS adapter ligation step. The AS adapters contain barcode sequences to identify samples, and thus we can combine samples to sequence in one lane of Illumina HiSeq 2000. Each ligation was performed in 30 µl reactions containing DNA, 15 µl 2× Quick Ligase buffer, 1 µl of 1 µM AS adapter and 1.5 µl Quick Ligase. The final PCR product was ∼125 bp and was extracted from 3% low molecular weight agarose gel electrophoresis and purified by Mini-elute gel extraction kit (Qiagen, Germany). Purified products were analyzed by a bioanalyzer followed by Illumina sequencing.

### Bioinformatic analysis of 5-mC and 5-hmC

We used an Illumina HiSeq 2000 at the institutional Epigenomics Shared Facility to sequence the libraries. Images generated by the Illumina sequencer were analyzed by Illumina pipeline software (versions 1.3 and 1.4). Initial data processing was performed using the default read length of 36 bp, after which we isolated the sequences in which we found adapter sequences on the 3′-end, replaced the adapter sequence with a poly (N) sequence of the same length and re-ran the Illumina ELAND pipeline again on these sequences with the sequence length set at 27 bp (the 2–28 bp subsequence). The data within the ELAND_extended.txt files were used for counting the number of aligned sequences adjacent to each CCGG (HpaII/MspI) site annotated in the hg19 freeze of the human genome at the UCSC genome browser. We permitted up to two mismatches in each sequence, and allowed a sequence to align to up to a maximum of 10 locations within the genome. For nonunique alignments, a sequence was assigned a partial count for each alignment location amounting to 1/n, where n represents the total number of aligned positions. To normalize the data between experiments, the number of sequences associated with each HpaII site was divided by the total number of sequences (including partial counts) aligning to all HpaII sites in the same sample.

As preformed previously, both 5-mC and 5-hmC values were depicted as values between 0 and 100 based on arctangent calculation (13). Transformation of the data to the angle measure substantially improves the correlation with bisulfite Mass Array validation data (13). Normalization of HpaII by MspI counts was done by plotting the MspI count on the x-axis and HpaII count on the y-axis for each site and the angle if calculated. This allows normalizing HpaII counts in terms of variability of the MspI representation. Hypomethylated loci were associated with relatively greater HpaII counts and a larger angle, whereas methylated loci will be defined by smaller angle values. Similar strategy was used to compare GT-MspI counts with MspI counts to determine a value for hydroxymethylation.

### Annotating 5-hmC and 5-mC

5-methyl cytosine levels were assessed as previously (13). Briefly, HpaII and MspI comparison was used for 5-mC calculation and degrees of arctangent of HpaII/MspI angle of <20 was used a cutoff for 5-mC. For 5-hmC assessment, degrees of arctangent of β-GT+MspI /MspI angle of <50 was used as cutoff. For added stringency, genomic loci where normalized β-GT+MspI counts were more than HpaII counts were used as second criteria for 5-hmC determination. Loci that fulfilled both criteria were flagged as 5-hmC. The data have been deposited in GEO (GSE42723).

Unsupervised clustering analysis, three samples were grouped by similarity of the 5-hmC or 5-mC measurements by average linkage clustering algorithm using the statistical software package R (www.r-project.org/).

### RNA-seq

One microgram of total RNA was used to prepare RNA-seq library (poly A selection based) using Illumina TruSeq technology (Illumina, San Diego, CA, USA). The generated libraries were sequenced on Illumina Hi-Seq 2000 (100 bp long single end reads). The sequences were aligned to Human genome (hg19, UCSC genome browser).

### RNA-seq data analysis

Alignment to the human reference genome 19 was performed using GSNAP (14). GSNAP detects novel splice events and known splice junctions based on ENSEMBL GTF annotations for the reference genome. Assignment of reads to genes was performed by htseq-count (http://www-huber.embl.de/users/anders/HTSeq/doc/count.html), a component of the HTSeq python library (http://www-huber.embl.de/users/anders/HTSeq/doc/overview.html). Assignments were made using known transcripts in the organism's ENSEMBL GTF annotation file using the *union* strategy and alignments with a quality score <20 were excluded. Differential expression analysis between tumor and normal samples was performed by edgeR, a bioconductor package specifically for the analysis of replicated count-based expression data (15). Gene counts were normalized by the Trimmed Mean of M component method. Gene expression was corrected using a moderated binomial dispersion correction then an exact test was used to assess differential expression. The resultant *P*-values were adjusted for false discovery rate by using Benjamini and Hochberg’s approach and only adjusted *P*-values with <0.05 were considered statistically significant.

#### Affinity pull down and qPCR validations

Genomic DNA with 5-hmC was pulled down by method described previously (12). Ten picograms of the input and pulled down DNA were amplified using the Whole Genome Amplification kit (Sigma-Aldrich, MO, USA) according to the manufacturer’s instructions. Quantitative PCR was performed in triplicates as follows: 100 pg of the amplified DNA was added to a final reaction of 10 µl containing 1× SsoFast™ EvaGreen® Supermix (Biorad, CA, USA), 0.5 µM forward and reverse primers (Supplementary Table S1). PCR was performed on a CFX96 Touch™ Real-Time PCR Detection System (Biorad, CA, USA). The fold enrichment for 5-hmC in cancer cell lines over normal control was calculated as 2 (Input − Pull down)_cell line_ / 2 (Input − Pull down)_control._ The average of three experiments was plotted with the statistical significance indicated.

### Genomic annotations

Annotation of CpG islands, Refseq genes and repetitive sequences were downloaded from the UCSC genome browser public database (hg19). CpG shores were defined as 2000 bp flanking regions on upstream and downstream of a given CpG island (16). In addition, the genome was partitioned to intergenic, intron, exon and promoter regions. Promoter regions were defined as the 2 kb window centered on the transcription start sites (TSS) of Refseq genes. We classified CpG dinucleotides as promoter, intronic, exonic or intergenic based on their overlap with these predefined regions. In addition, we classified in the CpG dinucleotides as CpG island or shore overlapping.

### Integration of 5-hmC, 5-mC and gene expression

5-hmC and 5-mC loci were mapped relative to RefSeq transcripts expressed at different levels in pancreatic cells. RefSeq transcripts were divided into two bins based on gene expression level (10th and 90th percentile) and 5-hmC or 5-mC genomic loci reads falling in 10-bp bins centered on TSS or end sites. Mean 5-hmC levels for Refseq transcripts expressed at increasing quartile levels (0–25, 26–50, 51–75, 76–100 percentiles) were calculated for control and pancreatic cancer cells and shown as histograms. Correlation between gene expression and 5-hmC or 5-mC was analyzed by linear regression analysis where hmC/mC = β_0_β_1_*X, with X representing expression.

### Sequencing data and coverage

Multiplexing of HELP-GT libraries was done with eight libraries per lane. The average number of reads for all samples varied from 6 to 10 million HpaII/MspI reads per sample with an average depth of coverage between 6× and 11× for each CCGG site.

**Table:** 

	MspI	HpaII	β-GT+MspI
Average number of reads	6 176 462	10 388 052	8 894 473
Average coverage	6×	11.2×	8.8×

### Cell lines and tissues

The low-passage patient-derived cell lines Pa03C and Pa04C were generated at Johns Hopkins University (17). Cells were cultured in Dulbecco's modified Eagle's medium supplemented with 10% Fetal Bovine Serum (FBS) and 1% Pen-Strep. Cultures were tested to be free of mycoplasma by MycoAlert Mycoplasma Detection Kit (Lonza, Switzerland), and DNA fingerprinting was used to authenticate cell lines. Brain and kidney tissues were removed from NOD.Cg-*Prkdc^scid^ Il2rg^tm^^1^^Wjl^/*SzJ mice (The Jackson Laboratory, Bar Harbor, ME, USA). Tissues were snap frozen in liquid N_2_, powdered with mortar and pestle under liquid N_2_ and DNA was obtained by phenol-chloroform extraction.

### Measurement of 5-mC and 5-hmC levels by mass spectrometry

DNA hydrolysis was performed as previously described (8). Briefly, 1 μg of genomic DNA was first denatured by heating at 100°C. Five units of Nuclease P1 (Sigma-Aldrich, Cat # N8630, MO, USA) were added and the mixture incubated at 45°C for 1 h. A 1/10 volume of 1 M ammonium bicarbonate and 0.002 units of venom phosphodiesterase 1 (Sigma-Aldrich, Cat # P3243, MO, USA) were added to the mixture and the incubation continued for 2 h at 37°C. Next, 0.5 units of alkaline phosphatase (Invitrogen, Cat # 18009-027, CA, USA) were added, and the mixture incubated for 1 h at 37°C. Before injection into the Zorbax XDB-C18 2.1 mm × 50 mm column (1.8 µm particle size) (Agilent Cat # 927700-902, CA, USA), the reactions were diluted 10-fold to dilute out the salts and the enzymes. Samples were run on an Agilent 1200 Series liquid chromatography machine in tandem with the Agilent 6410 Triple Quad Mass Spectrometer. LC separation was performed at a flow rate of 220 µl/min. Quantification was done using a LC-ESI-MS/MS system in the multiple reaction monitoring mode.

## RESULTS

### β-Glycosyl transferase modification allows detection of 5-hmC from small amounts of DNA

**Figure 1.**
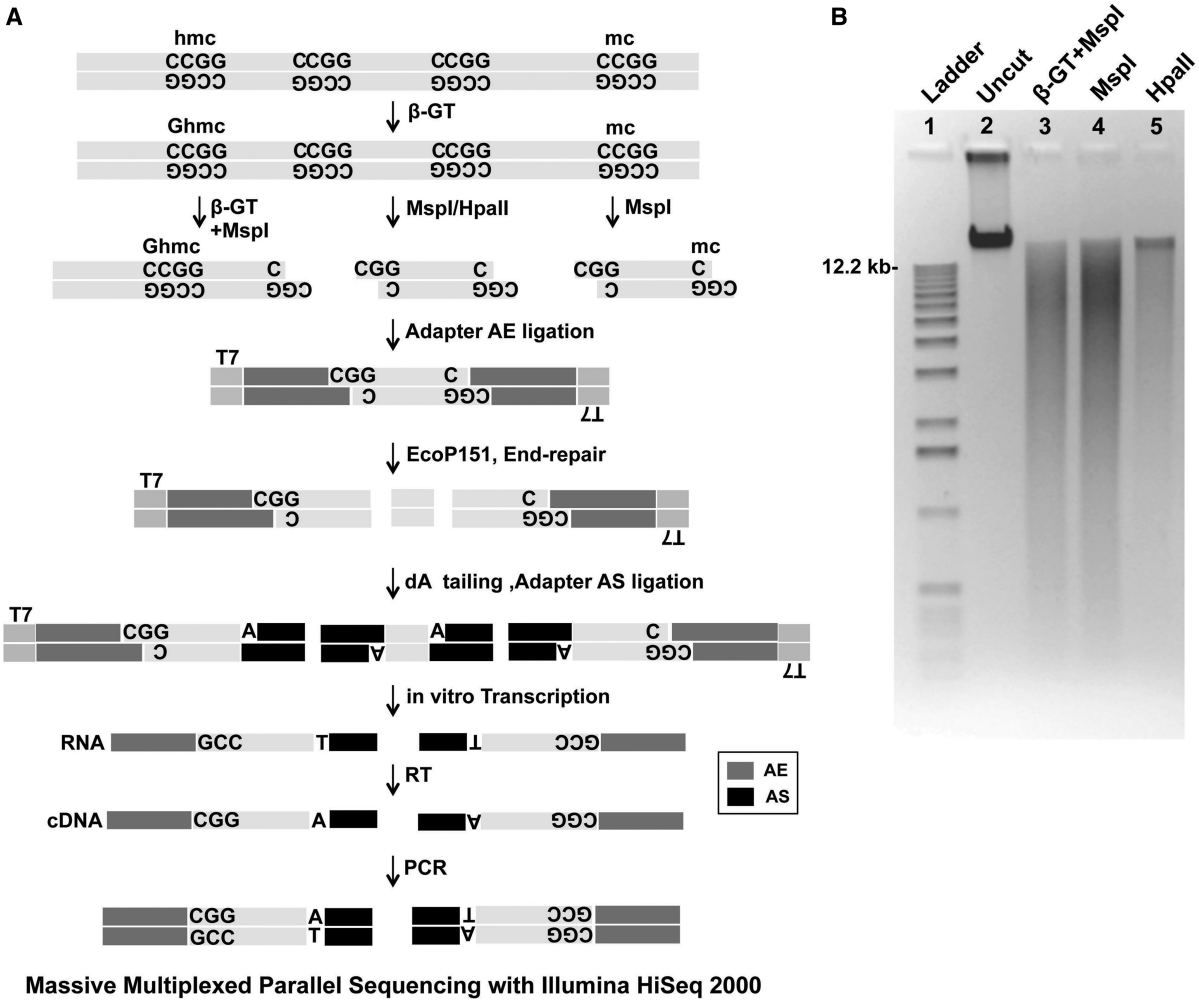
Schematic representation of the HELP-GT assay. Genomic DNA with or without β-GT pretreatment was digested by HpaII or MspI. HpaII only cuts at CCGG sequences where the central CCGG dinucleotide is unmethylated. The first Illumina adapter (AE) is ligated to the compatible cohesive end created, juxtaposing an EcoP15I site beside the HpaII/MspI digestion site and allowing EcoP15I to digest within the flanking DNA sequence as shown. An A overhang is created, allowing the ligation of the second Illumina adapter AS. This will create not only AE-insert-AS products but also AS-insert-AS molecules. By performing a T7 polymerase-mediated *in vitro* transcription from a promoter sequence located on the AE adapter, we can selectively enrich for the AE-insert-AS product, following which limited PCR amplification is performed to generate a single-sized product for Illumina sequencing (RT, reverse transcription). The final library was sequenced by multiplexing in-house adapter primers (in multiples of 4) using an Illumina HiSeq2000 (50 bp single end reads). Comparison of β-GT+MspI and MspI was used to determine hydroxymethylated sites. (**A**) Gel demonstrating decreased digestion by MspI after β-GT treatment of genomic DNA (**B**).

To develop a genome-wide assay that could simultaneously detect methyl cytosine (5-mC) and hydroxymethyl cytosines (5-hmC), we used the ability of β-GT to add a glucosyl group to the hydroxyl radical on the 5-hmC sites in the genome. It has been well described that treatment with β-GT and addition of glucosyl group to 5-hmC protects MspI sites (CCGG) from enzymatic digestion (12,18). It is previously known that HpaII and MspI are isoschizomers that act on the same site, although HpaII is methylation sensitive and cannot digest methylated CCGG sites. Using this concept we modified our HELP-tagging protocol (13) to incorporate a β-GT treatment step before digestion with methylation-sensitive enzymes. One microgram of genomic DNA was divided into three 300-ng aliquots and one of them was treated with β-GT. The rest of the samples were incubated in parallel as mock controls. MspI was then added to one of the mock controls and the β-GT digested sample (β-GT+MspI), while HpaII was added to the other mock control (Figure 1A and B). We hypothesized that comparing digestion patterns between HpaII and MspI would generate a list of 5-mC loci, while that between MspI with β-GT+MspI would yield a list of 5-hmC sites (Figure 1B). Samples were then ligated with adapters, digested with EcoP151 and libraries prepared as per our previous HELP tagging protocol (13). Reads were generated by multiplexed sequencing of libraries and the data were analyzed by a previously published algorithm (Supplementary Figure S1) (13) to yield reproducible numbers of 5-hmC and 5-mC loci in replicates (Supplementary Figure S2).

**Figure 2.**
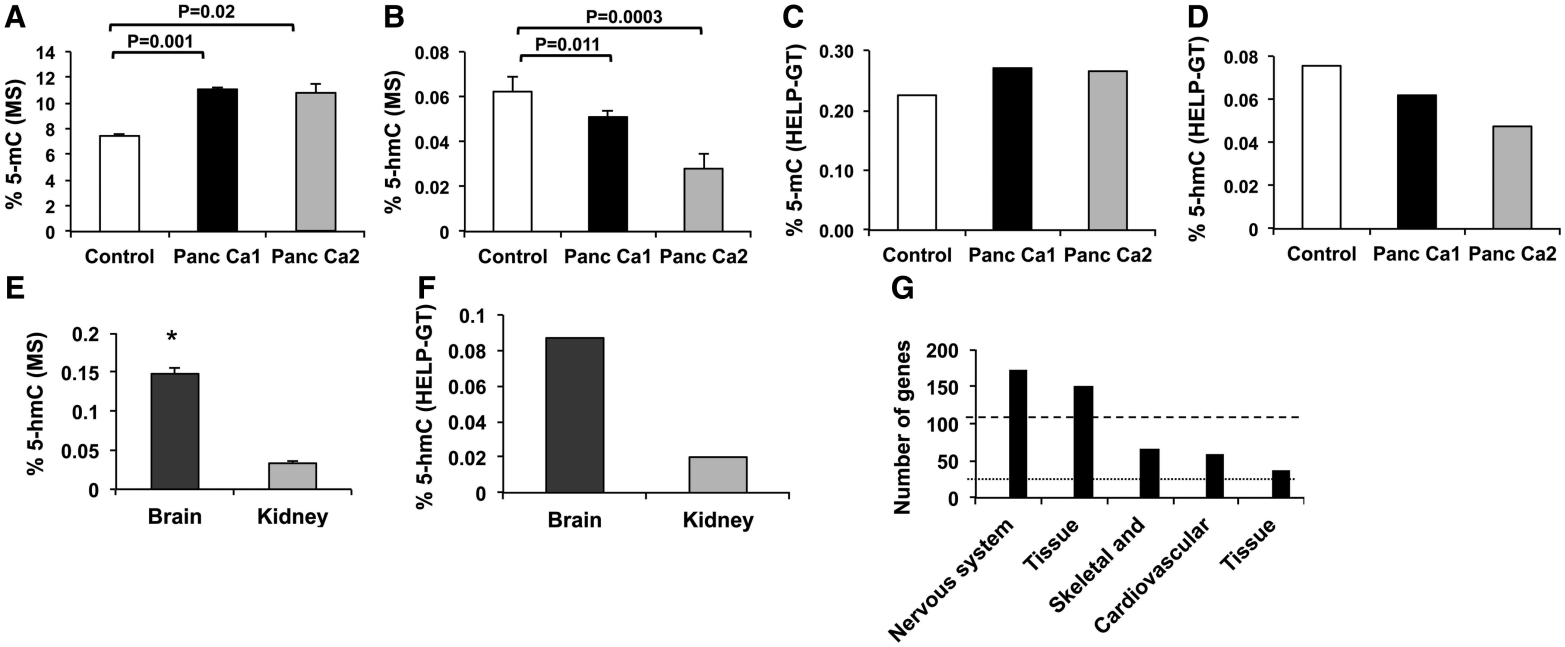
LC-MS and HELP-GT show similar global hydroxymethylation profiles in human and murine samples. (**A**) 5-mC and (**B**) 5-hmC percentages were measured by LC-MS for control (HPNE) and Panc Ca1 and Panc Ca2 cells. (Data from two experiments are shown ± SEM, *t* test, *P < 0.05). HELP-GT analysis shows similar proportion of 5-mC (**C**) and 5-hmC (**D**). Similarity in 5-hmC profiles was observed by LC-MS (*t* Test, *P < 0.05) (**E**) and HELP-GT (**F**) for murine brain and kidney tissues. 5-hmC loci in murine brain cells revealed enrichment for nervous system gene on Ingenuity pathway analysis (**G**).

We tested two pancreatic cancer cell lines [Panc Ca1 (Pa04C) and Panc Ca2 (Pa03C)] with immortalized healthy pancreatic cells (HPNE) as controls for these assays (17). These cell lines were also used for sensitive mass spectrometry based method for total 5-hmC and 5-mC determination. We observed that the total 5-mC content was increased in pancreatic cancer cells, consistent with previous reports showing hypermethylation in these tumors (19) (Figure 2A). Pancreatic cancer cells also had exhibited decreased 5-hmC levels when compared with control (Figure 2B). Analysis of 5-mC and 5-hmC by HELP-GT also yielded similar patterns (Figure 2C and D). The number of 5-hmC sites was determined by presence of methylated loci (based on HpaII/MspI) that furthermore had decreased ratio of β-GT+MspI–treated sample to the MspI control. Further stringency was imposed by only considering loci that had a greater number of normalized β-GT+MspI counts when compared with HpaII counts. We observed 139 338 unique 5-hmC sites in healthy pancreatic control cells when compared with 66 398 and 45 567 sites in the Panc Ca1 and Panc Ca2 cells, respectively. These results were similar to those seen by Mass Spec (results shown as percentages, Figure 2B and D).

We also tested the ability of this assay to interrogate murine tissues with relatively high amounts of 5-hmC in previous studies. Murine brain and kidney samples have been shown to be particularly enriched in 5-hmC content (12) and were tested for 5-hmC levels by both LC-MS and HELP-GT assay. We observed a good correlation between the results of these assays (Figure 2E and F) and observed 61 670 and 12 701 unique 5-hmC sites in brain and kidney tissues, respectively. Further locus-specific analysis revealed that brain 5-hmC sites significantly marked genes involved in nervous system development-specific gene pathways (Figure 2G) pointing to the biological validity of the 5-hmC loci flagged by the HELP-GT assay.

**Figure 3.**
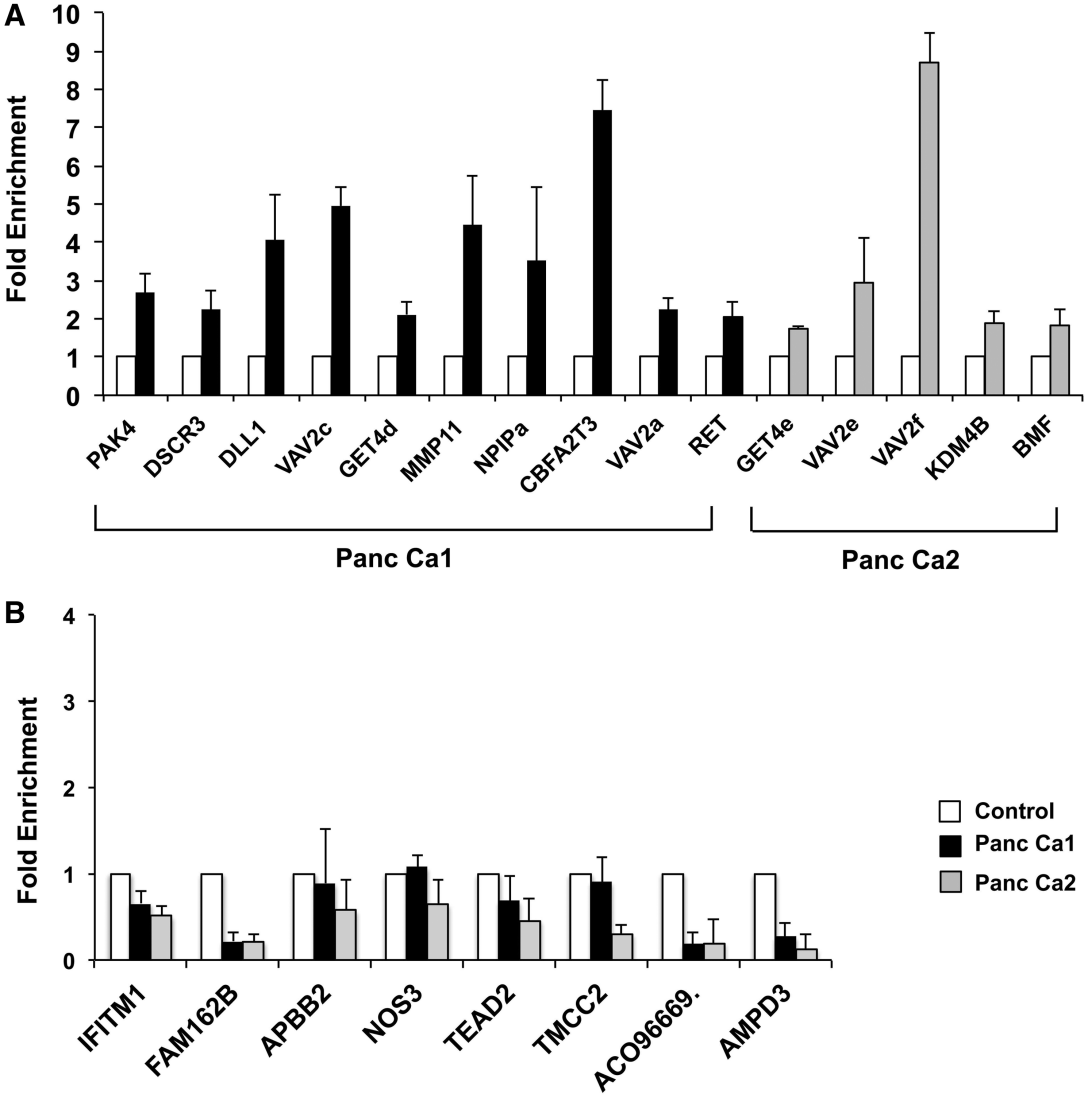
Hydroxymethylation validation by qRT-PCR. Genomic DNA from control and pancreatic cancer cells was glycosylated, biotinylated by click chemistry and affinity purified. qPCR for sites flagged as 5-hmC in pancreatic cancer by HELP-GT analysis showed enrichment for 5-hmC (**A**). Sites that were flagged as 5-mC with no 5-hmC by HELP-GT did not show any enrichment by qPCR (**B**).

We then proceeded to perform locus-specific validations of the 5-hmC sites. For validation, we used an established method that relies on biotin-aided pull down of 5-hmC sites (12). Briefly, genomic DNA was treated with β-GT followed by the use of click chemistry for biotin-avidin–mediated pull down of 5-hmC sites. Quantitative real-time polymerase chain reaction (qRT-PCR) analysis on the pull-down DNA revealed 5-hmC enrichment at the sites recognized by the HELP-GT assay (Figure 3A) for both cancer cell lines. Methylated sites without 5-hmC did not reveal any enrichment on validation (Figure 3B).

### Hydroxymethylation correlates positively with gene expression

**Figure 4.**
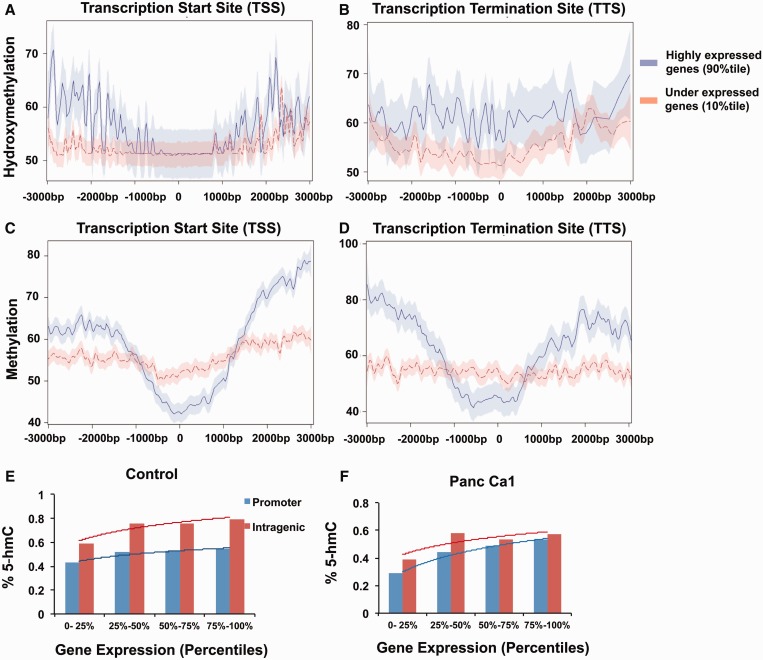
5-hmC correlates positively with gene expression at proximal and intragenic regions. 5-hmC and 5-mC loci were mapped relative to RefSeq transcripts expressed at different levels in pancreatic cells. RefSeq transcripts were divided into two bins based on gene expression level and 5-hmC or 5-mC genomic loci reads falling in 10-bp bins centered on TSS or end sites. Proximal and intragenic enrichment of 5-hmC is seen in highly expressed genes (**A** and **B**). 5-mC levels (shown with 95% confidence intervals) are decreased around TSS and enriched in intragenic areas for highly expressed genes. (**C** and **D**) Mean 5-hmC levels for Refseq transcripts expressed at increasing levels are shown for control and pancreatic cancer cells and show correlation with expression at both promoters and intragenic regions. Trend line based on Log regression (**E** and, **F**).

Next we wanted to determine the correlation of hydroxymethylation and methylation with gene expression. Results from RNA-seq performed on these cells were correlated with these epigenetic modifications. We observed a strong positive correlation between 5-hmC and gene expression at both proximal and intragenic regions (Figure 4A and B) [linear regression analysis, coefficient of correlation = 2.7; *P* < 0.001 for proximal (TSS ± 1 kb), and coefficient = 1.6, *P* < 0.001 for intragenic region (TSS to TTS)]. We observed that highly expressed genes had increased 5-hmC at both promoters as well as gene bodies (GB) (Figure 4A, B, E, F; Supplementary Figure S3). 5-mC correlated inversely with expression at the proximal regions with decreased amounts of 5-mC near the TSS of highly expressed genes (coefficient of correlation = −5.2, *P* < 0.001). Conversely, highly expressed genes were associated with increased 5-mC in intragenic regions (coefficient, *P* = 0.11), as has been reported in previous genome-wide analysis (16).

### 5-hmCs are redistributed in pancreatic cancer and occur in specific genomic locations

**Figure 5.**
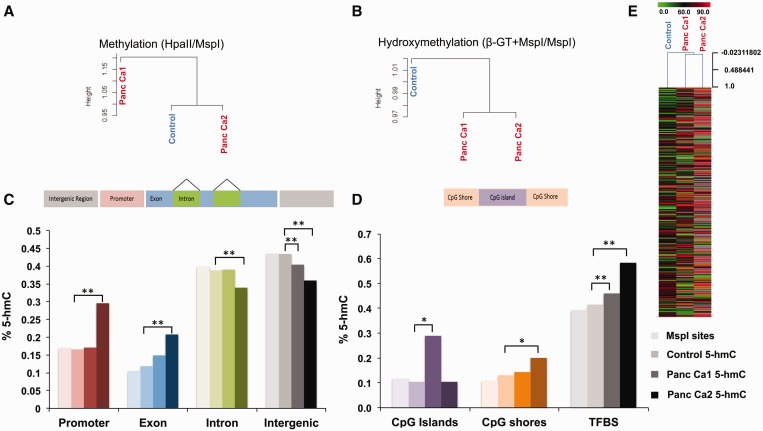
Redistribution of 5-hmC is seen in pancreatic cancer cells. Unsupervised clustering based on 5-hmC and 5-mC show that 5-hmC patterns can discriminate between control and pancreatic cancer samples (**A** and **B**). The relative distribution of 5-hmC sites at various genomic sites shows significant enrichment at promoters and exons in cancer (Test of proportions, *P* <0.05*, <0.01**) (**C**). Enrichment is also seen at TFBS and to a lesser extent at CpG islands and shores (**D**). Heatmap shows acquisition and loss of 5-hmC at various loci in cancer cells (**E**).

5-hmC marks were present throughout the genome in pancreatic control and cancer cells as has been seen in studies done in ES cells (Supplementary Figure S5) (12). Unsupervised clustering based on 5-hmC loci revealed that pattern of distribution of 5-hmC sites in both cancer samples were strikingly different from control cells (Figure 5B and E). This was in contrast to 5-mC loci that did not show differences between neoplastic and control cells on unsupervised clustering (Figure 5A). Comparison of control and cancer cells revealed that even though the total numbers of 5-hmC loci were decreased in cancer (Figure 2), there was a relatively higher enrichment of 5-hmC loci in specific regions of the genome. Specific enrichment of 5-hmC in exonic regions was seen in both cancers, with increasing percentage of 5-hmC sites seen in promoter regions in one cancer cell line (Figure 5C). The relative distribution of 5-hmC in control pancreatic cells was remarkably similar to the distribution of MspI sites (Supplementary Table S2) in the genome reflecting pervasiveness of this mark in benign cells. Increased relative 5-hmC was also seen in CpG islands and CpG shores Panc Ca1 and Panc Ca2, respectively (Figure 5D). Most interestingly, significant relative enrichment of 5-hmC was seen in transcription factor binding sites (TFBS) in both cancer samples (Figure 5D) demonstrating potential regulatory roles of this mark in the genome.

### Redistribution of 5-hmC in cancer affects oncogenic pathways

**Figure 6.**
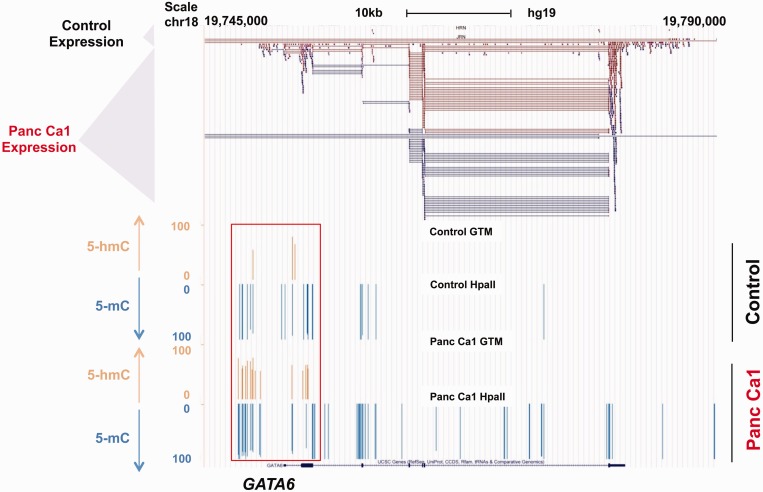
Acquisition of 5-hmC markers at oncogene promoters. The *GATA6* promoter has increased 5-hmC at promoter regions as shown by brown marks. 5-mC marks are shown as downward blue lines and are proportional to amount of 5-hmC. The scale is from 0 to 100 and represents quantitative values based on angles. A value of 0 represents no 5-mC, while 100 represents complete methylation. The top panel shows RNA-seq data demonstrating increased expression of *GATA6* in pancreatic cancer cells.

Next, we wanted to analyze the gene-specific distribution of 5-hmC in pancreatic cancer. We saw that even though total 5-hmC content is slightly decreased in cancer, there is widespread redistribution seen in cancer. Correlation with RNA-seq data revealed that important genes that were upregulated in cancer were associated with acquisition of 5-hmC marks in promoters and GB. Analysis of overexpressed genes and acquisition of 5-hmC marks revealed significant enrichment for cancer-associated pathways (Table 1). Examples included *GATA6* oncogene that showed an increased promoter 5-hmC in cancer when compared with control (Figure 6). *GATA6* has been shown to be frequently overexpressed in pancreatic cancer, but is amplified in only a minority of cases. Our data show that even though the promoter appears to be hyper methylated in cancer cells by conventional epigenomic analysis, it is in fact hydroxymethylated and correlates with the increased expression seen in the neoplastic cells. Similar 5-hmC enrichment around other oncogenic genes (Supplementary Figures S4A–C) revealed further the potentially important role of 5-hmC redistribution in cancer.

**Table 1.** Gene pathways upregulated and hydroxymethylated in pancreatic cancer

Disease	Pathways
Cancer	AATK,ABP1(includesEG:26),ADAMTS10,ADCY2,ALOX5,APBA2,ASCL2,BAI1,BMP7,BMPR1B, BRSK2(includesEG:100334759),CACNA1D,CACNA1S,CBLC,CEACAM6CENPF,CHST8,CNR1CNTNAP2, COL2A,CREB3L3,CSH1/CSH2,CYGB,DPEP3,DYNC1I1,EPHB2,ITGB4,FCGBP,FCN3,FLT4,FOXL2, FZD9,GABBR2,GNAS,GRIN2B,HDAC4HOXA9,IGFBP5IGSF9,ITGB4,JAG2,JPH3,KISS1,KLHL29, KLK11,KRT16,MATN3,MN1,NPAP1NR5A1,NRN1,NTRK3,OBSCN,PDCD6,PODN,PRKCZ,PRRX2, PTGER3,QPCTRASA4/RASA4B,RNF144A,SDK1,SIGLEC1,SLC12A7,SLC15A1,SLC6A3,SPOCK2, STAB1TGM2(includesEG:21817),TIMP3,TMC6,TNK2,TRIM29,TRPM8,USP2,VSTMLWFS1,ZNF217
Reproductive system disease	ABP1(includesEG:26),ADCY2,ASCL2,BMP7,BMPR1B,CEACAM6,EPHB2,FLT4,FOXL2,GNAS,GRIN2B, HOXA9,ITGB4,JAG2,KISS1,KLHL29,KLK11,NR5A1,PRKCZ,RAS4/RASA4B,RNF144A,SIGLEC1, SLC12A7,SPOCK2,TGM2(includesEG:21817),TIMP3,TMC6,TRPM8,USP2,VSTM2L,ZNF217
Skeletal and muscular disorders	ACTN2,ADAMTS10,BAIAP2,BMPR1B,CACNA1S,CAMK2B,CNR1,COL2A1,CPLX2,DYNC1I1,EPHB2, F8A1(includes others),GABRG3,GNAO1,GNAS,GRIN2B,HDAC4,HOXD13,ITGB4,KRT16,LMX1B, MATN3,PLA2G6,RASA4/RASA4B,RYR1,SLC6A3,SMAD6,SYNE2,TGM2(includes EG:21817),TIMP3,USP2
Hematological disease	ADAMTS10,BMPR1B,CACNA1S,CNR1,COL2A1,FBP1,FOXL2,GABRG3,GLP1R,GNAS,HDAC4,HOXD13, ITGB4,KRT16,LMX1B,MATN3,PKP1,RELN,RYR1,SYNE2,TIMP3

## DISCUSSION

We have modified the HELP-tagging assay to provide simultaneous determination of both 5-mC and 5-hmC in the genome with low amounts of DNA and with the use of limited sequencing resources. This assay provides a genome-wide survey of both 5-hmC and 5-mC sites of the genome and can be used to analyze large sample cohorts with modest sequencing resources. Furthermore, we show that this assay uses modest sequencing resources, can be used in different species and is validated at the global as well as single locus level by other methods (Supplementary Table S3). Overall our assay is able to provide single base-pair resolution analysis of ∼1 million sites in the human genome with the use of 1 μg of genomic DNA.

Our data from HELP-GT assay also reveals features of the hydroxymethylome of cancer, showing widespread novel redistribution of 5-hmC sites at specific genomic locations. Earlier reports based on immunohistochemistry revealed decreased 5-hmC in lung and prostate cancers (20) and our data also demonstrated decreased numbers of 5-hmC loci in pancreatic cancers. In spite of the absolute decrease in the 5-hmC levels in cancer, we observed enrichment of this modification at certain oncogenic gene promoters. These loci appear hyper methylated when tested only by HpaII/MspI representations. Examination using our β-GT approach revealed instead that these promoters have accumulated 5-hmC. *GATA6* is an oncogene that is involved in pancreatic cancer invasion and is overexpressed in nearly all pancreatic tumors. The mechanism of its overexpression was unclear as it is amplified in only 20% of cases (21,22), and the promoter of this gene was found to be hypermethylated in earlier studies, leading to the assumption that its expression is not controlled by DNA methylation. Our data show that the *GATA6* promoter appears methylated because standard techniques do not discriminate between methyl and hydroxymethylcytosine, and the *GATA6* promoter has acquired 5-hmC during transformation that correlates with its increased expression.

Overall, our data show striking correlation of 5-hmC with gene expression. This is observed near the TSS as well as in intragenic regions. This is in contrast with 5-mC, which is found to be decreased near TSS sites in promoter regions of highly expressed genes (12,16). The increased 5-mC that occurs in the GB of highly expressed genes is also accompanied by increased 5-hmC. These results demonstrate that estimating 5-hmC is important for evaluating the effect of DNA methylation on gene expression and should be measured in future epigenomic studies in cancer and other diseases. Taken together, our data show that the HELP-GT assay is a robust genome-wide survey assay that allows simultaneous high-resolution determination of 5-hmC and 5-mC loci from small amounts of DNA.

## SUPPLEMENTARY DATA

Supplementary Data are available at NAR Online.

## FUNDING

NIH [R01HL116336]; Immunooncology Training Program [T32 CA009173]; Gabrielle’s Angel Foundation, Leukemia and Lymphoma Society and Department of Defense. Funding for open access charge: NIH [R01HL116336].

*Conflict of interest statement*. None declared.

## Supplementary Material

Supplementary Data

Supplementary Data
