# Supplementary material for: Genome-wide hydroxymethylation tested using the HELP-GT assay shows redistribution in cancer
Source: Nucleic Acids Res. 2013 Jul 16;41(16):e157. doi: 10.1093/nar/gkt601 (PMC3763560; doi:10.1093/nar/gkt601)
Supplement: Supplementary Data [file supp_41_16_e157__index.html]

Genome-wide hydroxymethylation tested using the HELP-GT assay shows redistribution in cancer — Genome-wide hydroxymethylation tested using the HELP-GT assay shows redistribution in cancer — Supplementary Data 

# Genome-wide hydroxymethylation tested using the HELP-GT assay shows redistribution in cancer

## 

files

**Files in this Data Supplement:**

- Supplementary Data - pdf file
