# Supplementary material for: Genome-wide hydroxymethylation tested using the HELP-GT assay shows redistribution in cancer
Source: Nucleic Acids Res. 2013 Jul 16;41(16):e157. doi: 10.1093/nar/gkt601 (PMC3763560; doi:10.1093/nar/gkt601)

FIGURE LEGENDS

Figure S1. Schema of HELP-GT assay. HELP-tagging libraries were prepared by the process described previously (Suzuki et al. 2010) with some modifications as follows. Based on the assumption that most of the hydroxymethylated cytosines come from methylated cytosines, we added a step of β-GT prior to MspI or HpaII digestions (Song, 2010; Josse and Kornberg, 1962). The final library was sequenced by multiplexing in-house adapter primers using Illumina HiSeq 2000.

Figure S2. 5-hmC and 5-mC estimation by the HELP-GT assay are reproducible. Unsupervised clustering of two independent replicates shows high degree of concordance for both 5-hmC and 5-mC analysis (A, C). A high degree of concordance was also seen in gene specific localization of 5-hmC and 5-mC sites for replicates (B, D) as shown for the representative control sample.

Figure S3. 5-hmC correlates with gene expression at proximal and intragenic regions: 5-hmC and 5-mC loci were mapped relative to RefSeq transcripts expressed at different levels in pancreatic cancer (Panc Ca1) cells. RefSeq transcripts were divided into two bins based on gene expression level and 5-5-hmC or 5-mC genomic loci reads falling in 10-bp bins centered on transcription start sites or end sites. Proximal and intragenic enrichment of 5-hmC is seen in highly expressed genes (A, B). 5-mC levels are decreased around TSS and enriched in intragenic areas for highly expressed genes. (C, D).

Figure S4. Acquisition of 5-hmC at different promoters *MMP11* (A) *VAV2* (B**)** and *LATS2* (C) show increased 5-hmC at promoter regions as shown by brown marks. 5-mC marks are shown as downward blue lines. The scale is from 0 to 100. Top panel shows RNA-seq data demonstrating increased expression of *MMP11, VAV2 and LATS2* in pancreatic cancer cells.
lines. The scale is from 0 to 100. Top panel shows RNA-seq data demonstrating increased expression of *RBM38* in pancreatic cancer cells.

Figure S5. **5hmC loci are distributed throughout the genome** Circos plots of 5-hmC loci (orange bars) and gene expression (by RNA seq, green bars) show genome wide prevalence of 5-hmC loci in both control (A) and cancer cell lines Panc Ca1 (B) and Panc Ca2 (C). The length of the bars corresponds to amount of 5-hmC and magnitude of expression.

Table S1:

Genomic locations and primers for 5-hmC validations:

Chromosome	Position	Gene	Fwd primer	Rev primer
chr19	39658315	PAK4	CTTGGGACCAGCTGAGACAG	CCCAGAGCTAGGACCATCCA
chr21	38603328	DSCR3	GGACAAGTGTCCCACGTCAT	ACCTGTTCTCCTGGTCCTCA
chr6	170594425	DLL1	TGGGGTCACACTCGTCAATC	AGGGGAGCTACACTTGCTCT
chr9	136748793	VAV2c	CCCAAGTCATCCCCAGCC	ATTGCACAGGTGACCGGAAG
chr7	923330	GET4d	CCCTATCTGGAAGGCACGTC	GGTGACTAAGCCAGGACAGG
chr22	24111338	MMP11	TCTGGGTGACTTTGCAGTGG	AGCAGTGATGAGGGACAGT
chr16	15028924	NPIPa	GACGGTGCAGATGTCCCATA	TGCTCAGGACAGGGATGAGA
chr16	88990095	CBFA2T3	AGCAGCTGGGTTGTGAAGG	GTTCCAAAGCCGACCTCCAT
chr9	136746964	VAV2a	TACTACTCCAGGCAGCGTGT	ACTTGCTGCGTGTCTGATCT
chr10	43614886	RET	TGTGTCCACCCCCTTACTCA	CAGCTTGGGTCTTCCAGGAG
chr9	136844169	VAV2e	TTTAGGGTCTCCTCCAGCCA	ACGAAAGCTCTTCCGAAGCC
chr9	136845219	VAV2f	AGTAGGGTGGAGAACAGGCT	GAGACAGAGAACCAGGTGCC
chr7	922846	GET4e	GTGACTCCTGGCAGAGCAG	ATTTCACAAGGACCCCTCCG
chr19	5041526	KDM4B	AGCCAGGCTTTCTCTGATTTT	GTTTGTAACACGCTGTGGGC
chr15	40396398	BMF	CAGCAGACTCAACCCTCCTC	TCAGTGCATTGCAGACCAGT

Table S2:

Genomic distribution of HpaII/MspI sites

**Genomic Position**	**Number of sites**
Total	2,326,002
CpG islands	288,413
CpG shores	255,321
Promoter	313,138
Gene Body	950,168

Table S3:

MspI, HpaII and βGT-MspI counts of Panc Ca1 samples for the loci that were validated to have 5hmC marks.

Gene Name	HpaII counts	GT-MspI counts	MspI counts
VAV2	1	1	3
CBFA2T3	4	3	14
MMP11	1	1	4
NPIP	3.285714286	3.142857143	8.714285714
PAK4	4	5	14
DLL1	1	5	14
RET	3	2	12
DSCR3	1	3	6
GET4	0.333333333	2.333333333	8.666666667

Figure S1

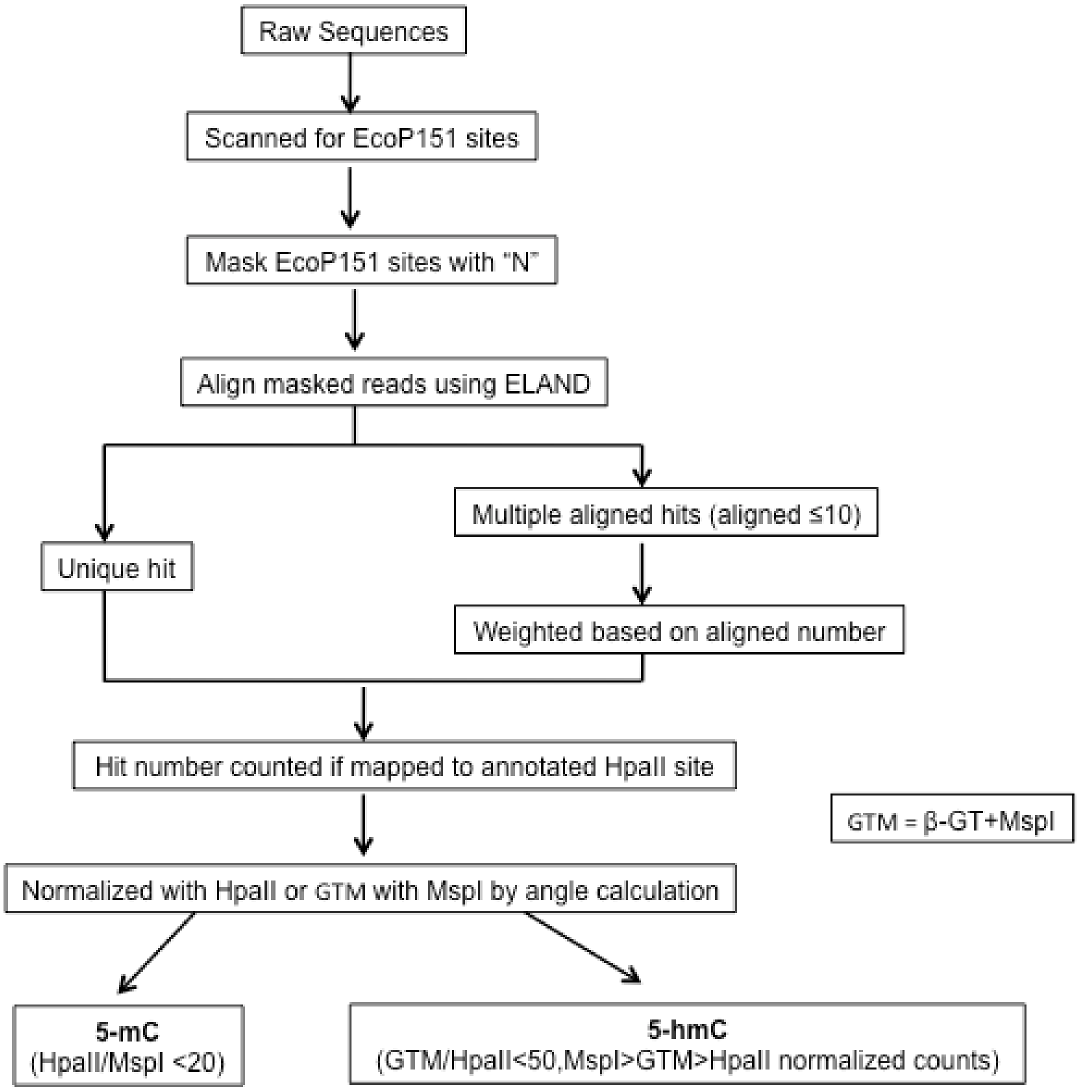
Raw Sequences
Scanned for EcoP151 sites
Mask EcoP151 sites with "N"
Align masked reads using ELAND
Unique hit
Multiple aligned hits (aligned ≤10)
Weighted based on aligned number
Hit number counted if mapped to annotated HpaII site
GTM = β-GT+MspI
Normalized with HpaII or GTM with MspI by angle calculation
5-mC
(HpaII/MspI <20)
5-hmC
(GTM/HpaII<50,MspI>GTM>HpaII normalized counts)

Figure S2

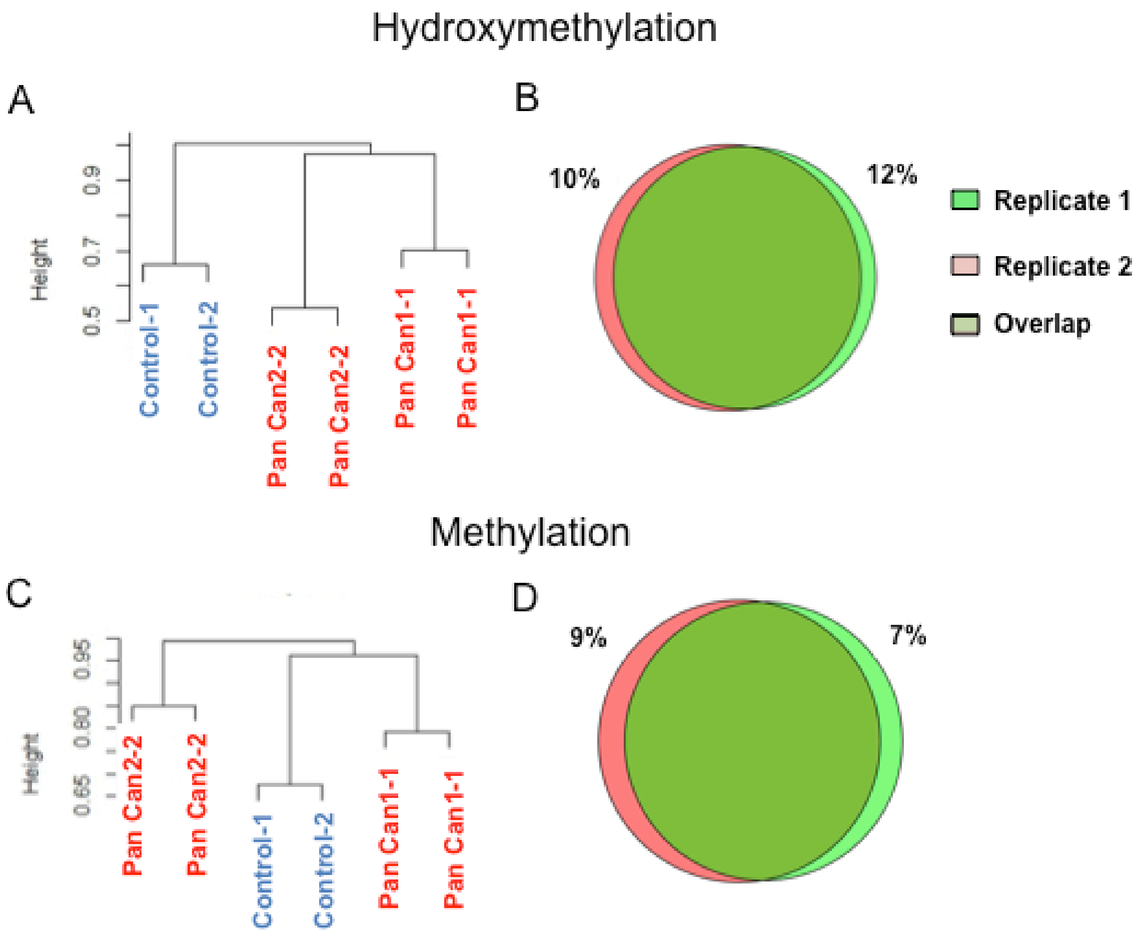

Hydroxymethylation
A
B
Height
0.5
0.7
0.9
Control-1
Control-2
Pan Can2-2
Pan Can2-2
Pan Can1-1
Pan Can1-1
10%
12%
Replicate 1
Replicate 2
Overlap
Methylation
C
D
Height
0.65
0.80
0.95
Pan Can2-2
Pan Can2-2
Control-1
Control-2
Pan Can1-1
Pan Can1-1
9%
7%

Figure S3

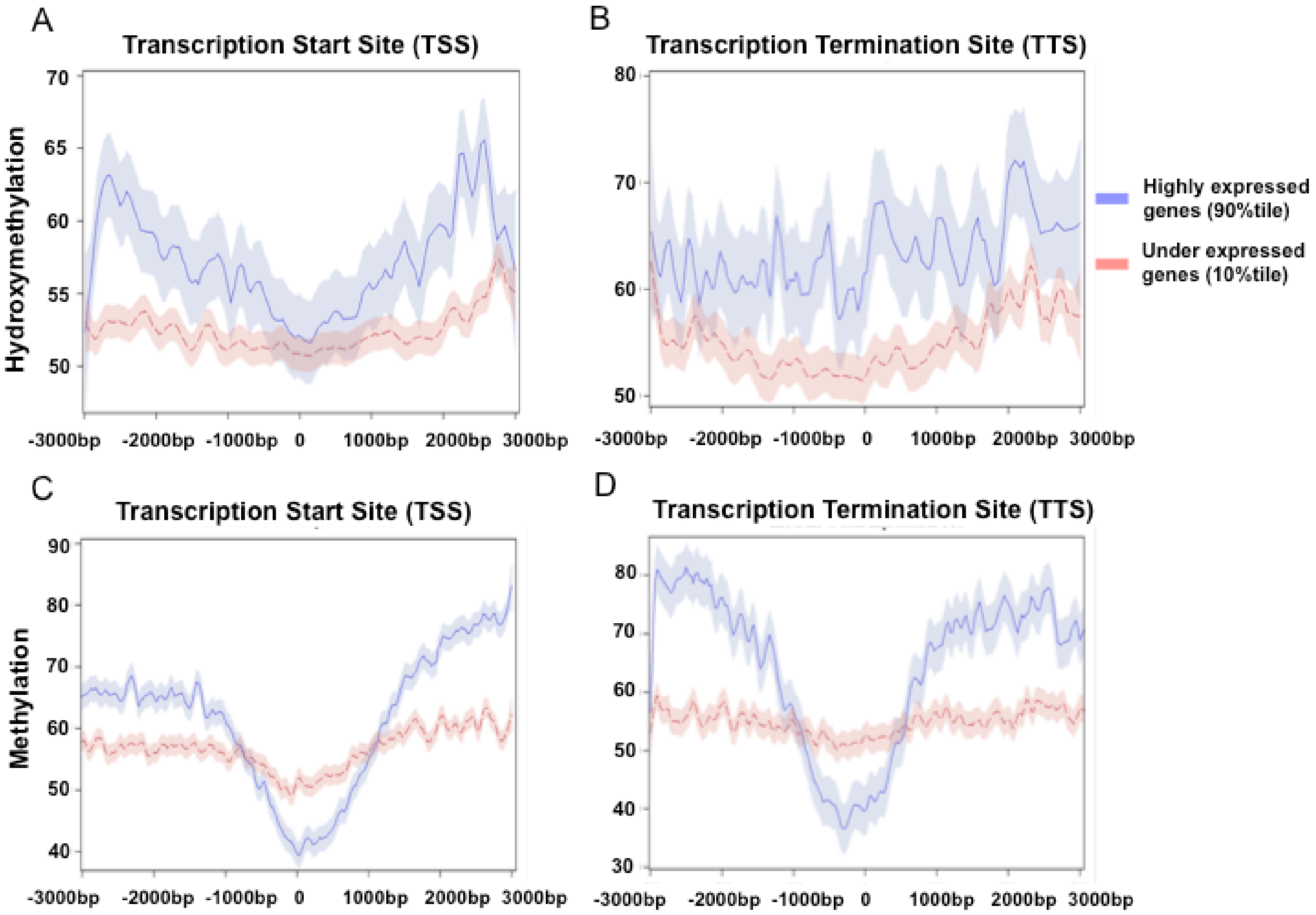
A
Transcription Start Site (TSS)
Hydroxymethylation
70
65
60
55
50
-3000bp
-2000bp
-1000bp
0
1000bp
2000bp
3000bp
B
Transcription Termination Site (TTS)
80
70
60
50
-3000bp
-2000bp
-1000bp
0
1000bp
2000bp
3000bp
Highly expressed genes (90%tile)
Under expressed genes (10%tile)
C
Transcription Start Site (TSS)
Methylation
90
80
70
60
50
40
-3000bp
-2000bp
-1000bp
0
1000bp
2000bp
3000bp
D
Transcription Termination Site (TTS)
80
70
60
50
40
30
-3000bp
-2000bp
-1000bp
0
1000bp
2000bp
3000bp

Figure S4

A

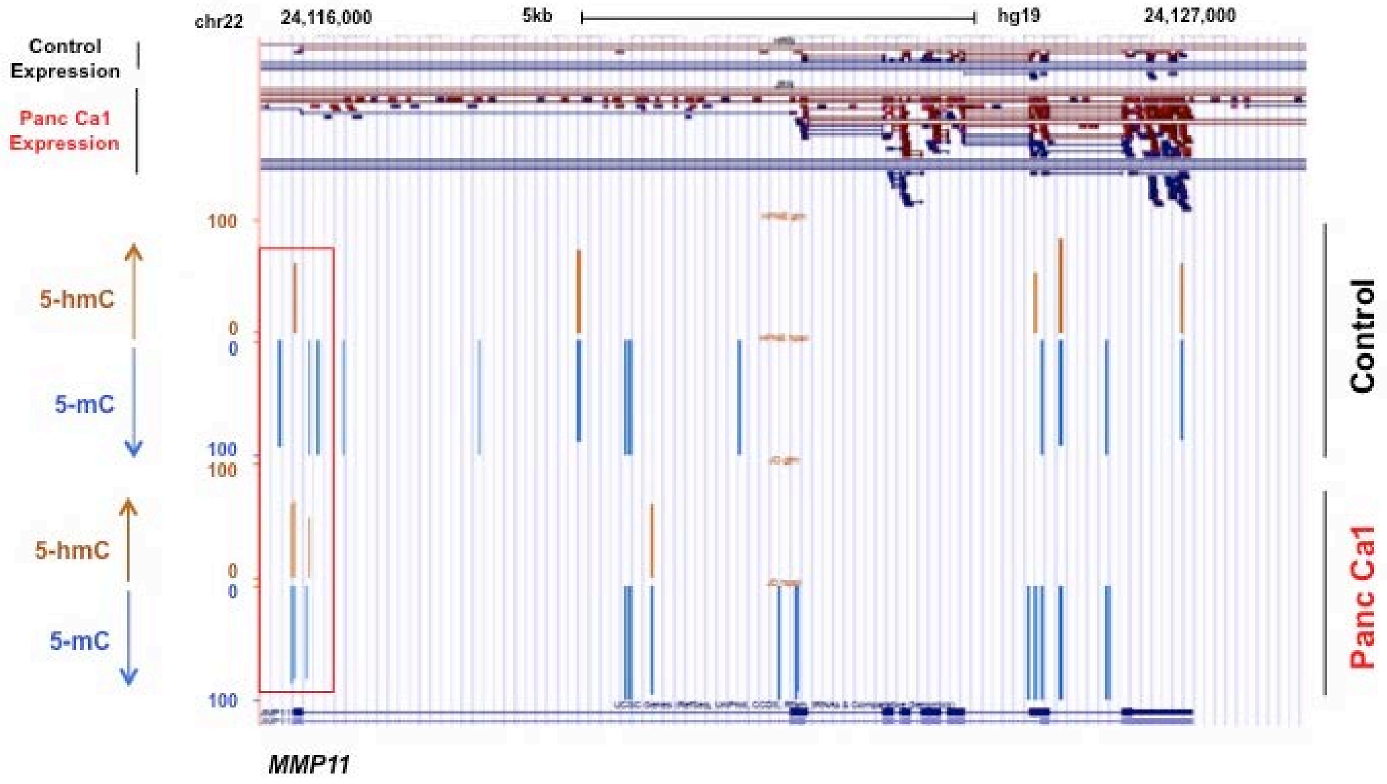
chr22
24,116,000
5kb
hg19
24,127,000
Control Expression
Panc Ca1 Expression
100
5-hmC
0
0
5-mC
100
100
5-hmC
0
0
5-mC
100
Control
Panc Ca1
MMP11

Figure S4

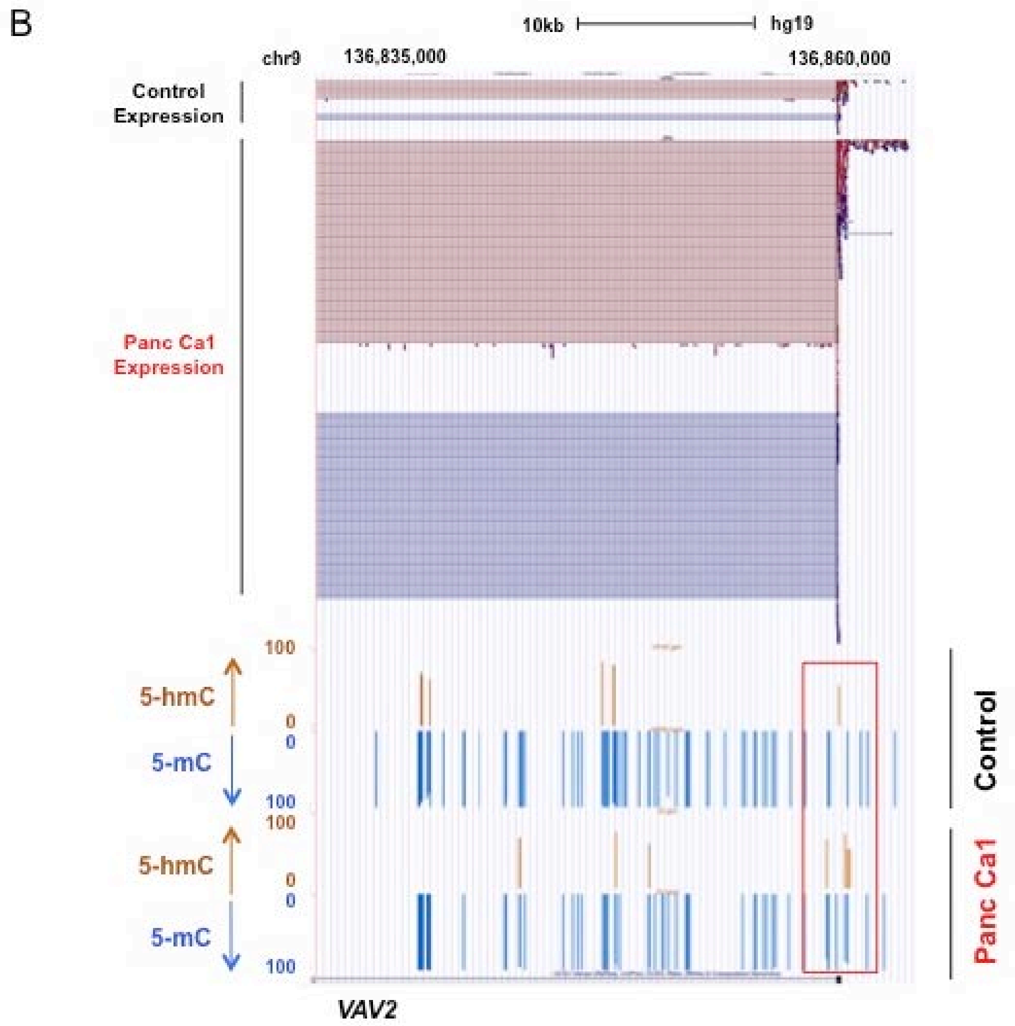
B
10kb
hg19
chr9
136,835,000
136,860,000
Control
Expression
Panc Ca1
Expression
100
5-hmC
0
0
5-mC
100
100
5-hmC
0
0
5-mC
100
Control
Panc Ca1
VAV2

Figure S4

C

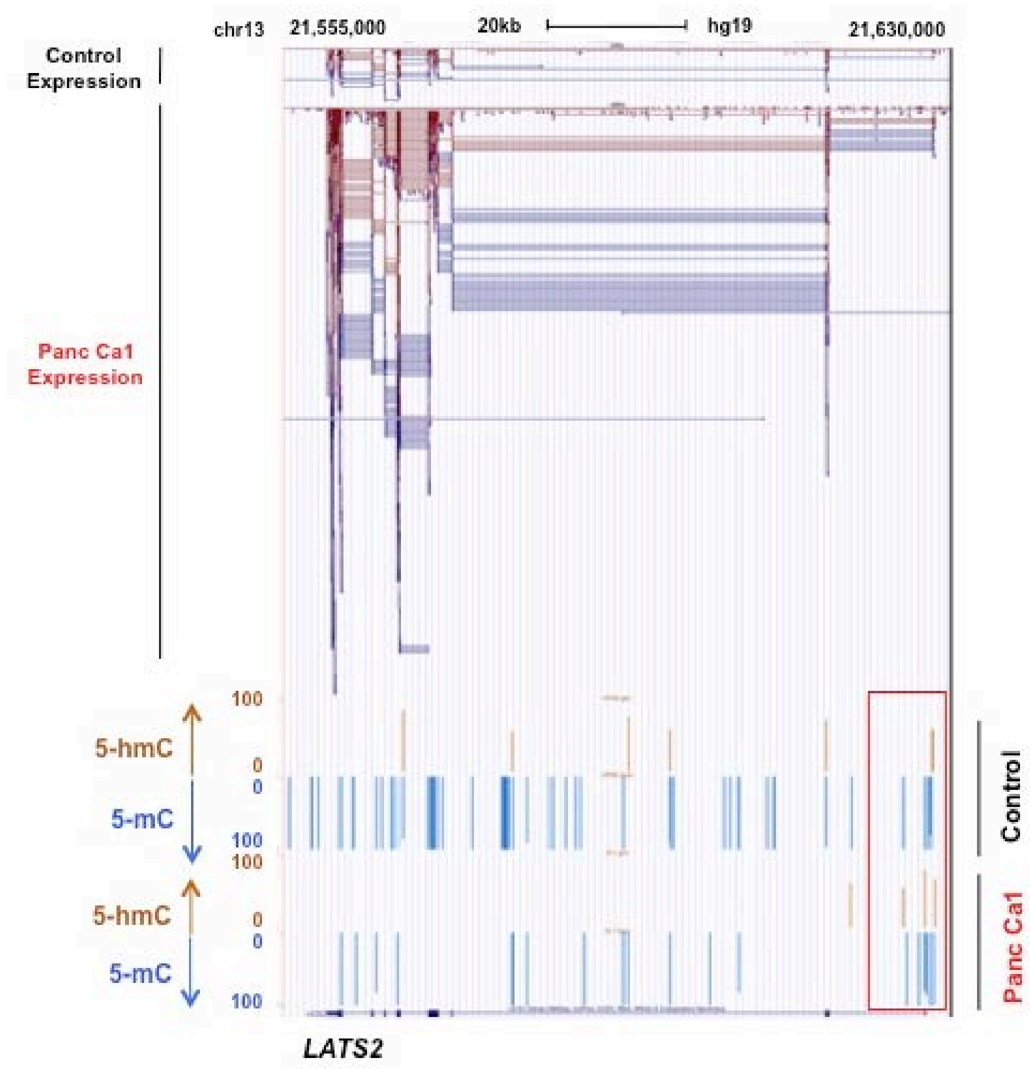

chr13
21,555,000
20kb
hg19
21,630,000
Control Expression
Panc Ca1 Expression
100
5-hmC
0
0
5-mC
100
100
5-hmC
0
0
5-mC
100
Control
Panc Ca1
LATS2

Figure S5

A

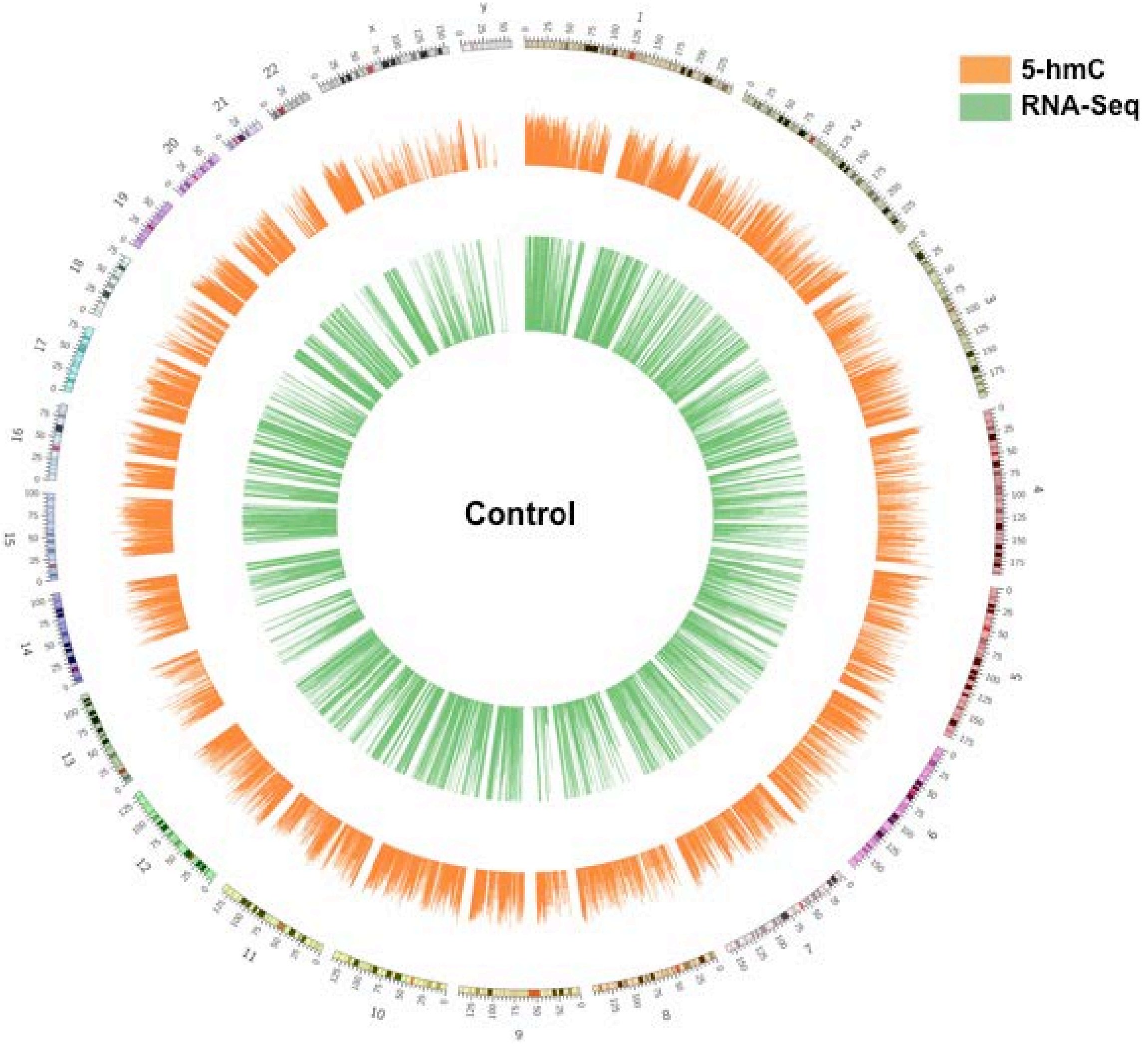

Figure S5

B

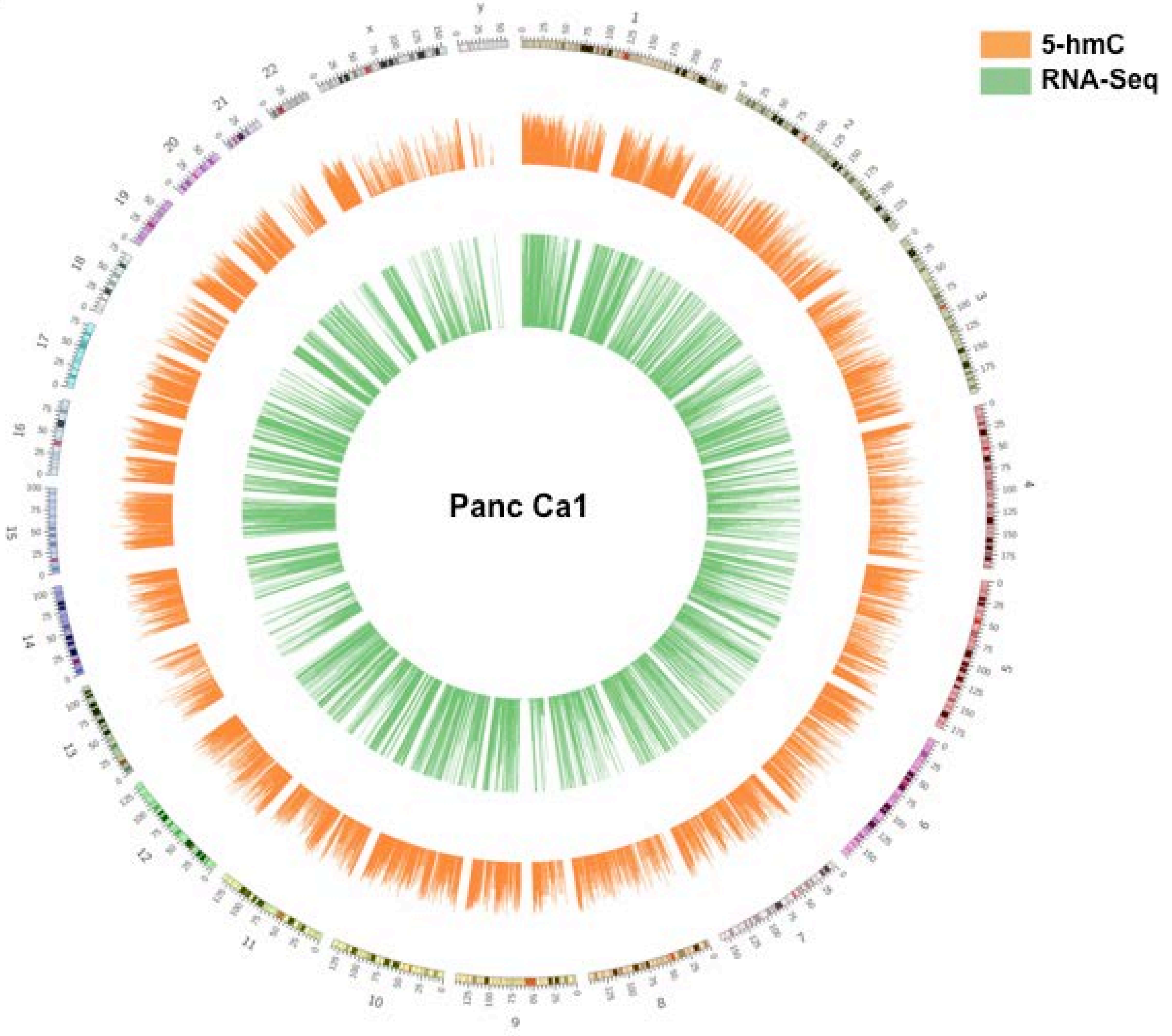

Figure S5

C

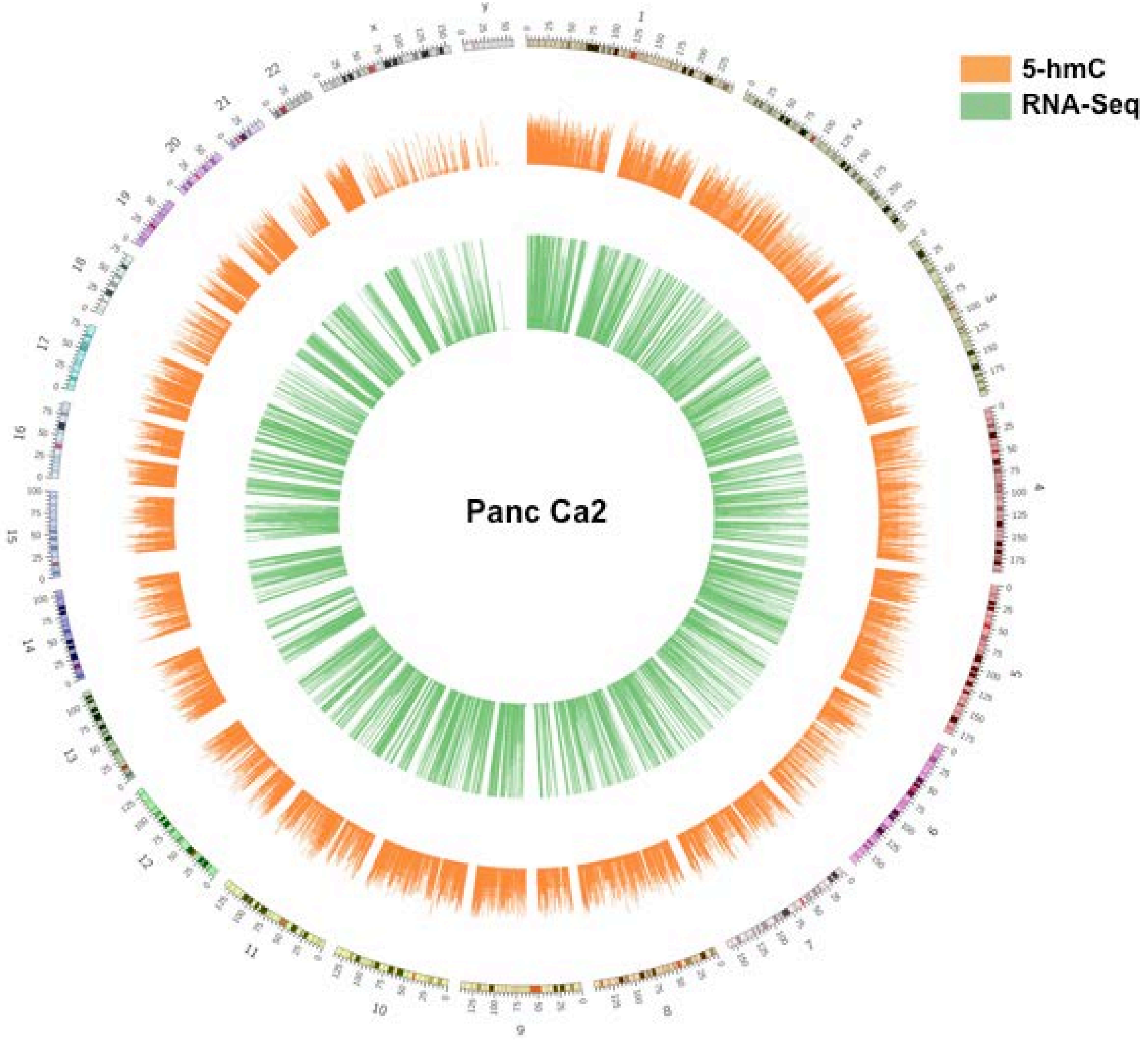

Supplement: Supplementary Data [file supp_gkt601_Supplemental_text_NAR_June_26.pdf]
